# The Transcription Factor REST Is Lost in Aggressive Breast Cancer

**DOI:** 10.1371/journal.pgen.1000979

**Published:** 2010-06-10

**Authors:** Matthew P. Wagoner, Kearney T. W. Gunsalus, Barry Schoenike, Andrea L. Richardson, Andreas Friedl, Avtar Roopra

**Affiliations:** 1 Department of Neurology, University of Wisconsin-Madison, Madison, Wisconsin, United States of America; 2 Molecular and Cellular Pharmacology Graduate Program, University of Wisconsin-Madison, Madison, Wisconsin, United States of America; 3 Program in Cellular and Molecular Biology, University of Wisconsin-Madison, Madison, Wisconsin, United States of America; 4 Department of Pathology, Brigham and Women's Hospital, Boston, Massachusetts, United States of America; 5 Department of Pathology and Laboratory Medicine, University of Wisconsin-Madison, Madison, Wisconsin, United States of America; The University of North Carolina at Chapel Hill, United States of America

## Abstract

The function of the tumor suppressor RE1 silencing transcription factor (REST) is lost in colon and small cell lung cancers and is known to induce anchorage-independent growth in human mammary epithelial cells. However, nothing is currently known about the role of this tumor suppressor in breast cancer. Here, we test the hypothesis that loss of REST function plays a role in breast cancer. To assay breast tumors for REST function, we developed a 24-gene signature composed of direct targets of the transcriptional repressor. Using the 24- gene signature, we identified a previously undefined RESTless breast tumor subtype. Using gene set enrichment analysis, we confirmed the aberrant expression of REST target genes in the REST–less tumors, including neuronal gene targets of REST that are normally not expressed outside the nervous system. Examination of REST mRNA identified a truncated splice variant of REST present in the REST–less tumor population, but not other tumors. Histological analysis of 182 outcome-associated breast tumor tissues also identified a subpopulation of tumors that lack full-length, functional REST and over-express the neuroendocrine marker and REST target gene Chromogranin A. Importantly, patients whose tumors were found to be REST–less using either the 24-gene signature or histology had significantly poorer prognosis and were more than twice as likely to undergo disease recurrence within the first 3 years after diagnosis. We show here that REST function is lost in breast cancer, at least in part via an alternative splicing mechanism. Patients with REST–less breast cancer undergo significantly more early disease recurrence than those with fully functional REST, regardless of estrogen receptor or HER2 status. Importantly, REST status may serve as a predictor of poor prognosis, helping to untangle the heterogeneity inherent in disease course and response to treatment. Additionally, the alternative splicing observed in REST–less breast cancer is an attractive therapeutic target.

## Author Summary

Breast cancer is a heterogeneous disease, with highly variable disease outcomes and responses to treatment for otherwise indistinguishable tumors. Understanding this heterogeneity holds the key to better determining disease prognosis and tailoring treatments to the tumors for which they will be most efficacious. Some of the most successful work dissecting the differences between histologically identical tumors with differing disease outcomes has come from profiling the array of protein-coding transcripts present in every tumor and dividing the breast cancer profiles into multiple subtypes of varying aggressiveness. Importantly, these profiles are now being used in the clinic to predict disease outcome and plan treatment. Using a similar molecular-profiling strategy, we have identified a previously unrecognized subset of breast cancers in which the tumor suppressor gene REST is lost, which display a highly aggressive disease course. Intriguingly, we have traced the loss of the tumor suppressor to the presence of a variant of the REST protein normally found in the brain following seizures, which represents a unique and attractive therapeutic target. Additionally, the gene signature used to identify REST–less tumors shows no overlap with the profiles currently used in the clinic to assess tumor aggressiveness and may be an important new diagnostic tool.

## Introduction

Identification of tumor suppressors is hampered by the fact that their loss of function can occur through any one of many mechanisms including inactivating mutations, aberrant splicing and copy number aberration. Transcription factor tumor suppressors control many downstream genes in a given cell; however, using the absence or presence of a downstream gene as a proxy for loss of tumor suppressor function is problematic because each gene is likely to be regulated by multiple transcription factors. Genome-wide transcription profiling has opened up the possibility of simultaneously measuring expression levels of multiple, if not all, downstream target genes of a tumor suppressor. In this report we describe the generation of such a signature for the tumor suppressor RE1 Silencing Transcription Factor (*REST* Enterez GeneID 5978), also known as Neuron Restrictive Silencing Factor (NRSF), and the identification of this signature in breast cancer.

REST was originally isolated from a screen for factors that confer neuron-restricted gene expression upon neuronal genes [1], [2]. REST represses transcription by binding to the 17–33 base pair Repressor Element 1 (RE1) found in the regulatory regions of target genes [3], [4]. Around 2,000 genes in the human and mouse genomes have been identified as direct targets of REST [4], [5]. REST represses transcription by recruiting chromatin modifying enzymes such as histone deacetylases (HDACs) and histone methyltransferases (HMTs) via the N-terminal and C-terminal repression domains respectively and possesses a DNA binding domain (DBD) consisting of 8 zinc fingers[6]–[10].

Alternative splicing of REST occurs in the mouse brain during seizures and results in the expression of the truncated splice variant, REST4 [11]. The inclusion of an alternative exon into the coding region of REST results in the insertion of a premature stop codon, generating a truncated protein containing just the N-terminal repression domain and five of the eight zinc fingers. REST4 is a poor repressor of transcription because of reduced DNA binding ability and loss of at least one repression domain [8].

Splicing of REST mRNA into its REST4 form also occurs in small cell lung cancer (SCLC), resulting in the overexpression of REST target genes and imparting a neuroendocrine phenotype on the cells [12]. Re-expression of functional REST in REST4-expressing SCLC cells induces apoptosis, suggesting that suppression of REST function is key to survival of these cells [13]. In an unbiased tumor suppressor screen, REST loss conferred anchorage-independent growth upon immortalized human mammary epithelial cells [14]. The authors described a truncating point mutation that eliminates the C-terminal repression domain in colon cancer, the expression of which was sufficient to induce anchorage-independent growth. However, no role for REST in breast cancer was reported.

To determine whether the tumor suppressor REST is lost in breast cancer, we developed a gene signature-based approach to screen for loss of REST function by measuring the expression of a cohort of REST target genes. Using this gene signature as well as an immuno-histochemical screen, we show here that an aggressive subset of breast cancers lack functional REST (“REST–less tumors”), instead, often expressing the truncated REST splice variant REST4.

## Results

### REST expression in BC tumors

Given that loss of REST induces anchorage-independent growth in mammary epithelial cells, and is lost in colon [14] and small cell lung cancers [12], we asked whether REST also plays a role in breast cancer. REST function is regulated at multiple post-transcriptional levels during differentiation and disease [15]–[18] and so we needed a method of detecting a loss of REST function in a tumor, regardless of the underlying mechanism responsible for the loss of the tumor suppressor. To accomplish this, we developed a gene signature comprised of direct targets of REST repression with which to interrogate tumor microarray datasets to search for a loss of REST function.

**Figure 1:**
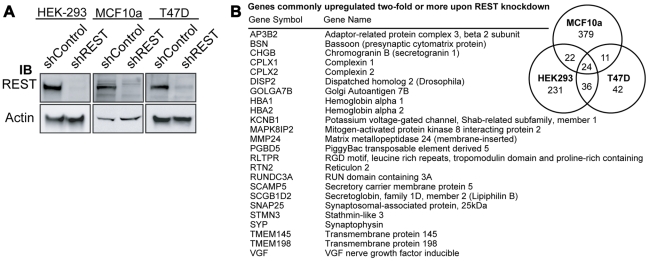
Generation of a 24-gene signature for loss of REST. (A) REST expression was knocked down in HEK, MCF10a, and T47D cell lines using lentiviral delivery of shRNA targeting REST (shREST) or a control non-targeting shRNA (shControl). Western blot analysis of total protein levels of REST and beta-actin was performed to confirm knockdown. (B) Microarray analysis of RNA from shREST and shControl cells for each cell type identified 24 genes (listed) that were up-regulated by at least 2 fold in all three lines.

There are approximately 2,000 RE1-bearing genes in the mouse and human genomes; however, not all of these genes are repressed by REST in every cell type [19], [20]. Therefore, in order to generate a gene signature with which to interrogate tumor microarrays for loss of REST function, we first sought to identify a set of REST target genes that are coordinately up-regulated when REST function is lost in multiple cell types, including tumorigenic and non-tumorigenic breast cells. To that end, we stably infected three disparate cell lines, the non-tumorigenic MCF10a mammary epithelial cell line, T47D breast cancer line and the unrelated human embryonic kidney-293 line with lentivirus expressing a non-targeting control shRNA (shCon) or an anti-REST shRNA (shREST), which resulted in the robust knockdown of REST protein (Figure 1A). RNA from shCon and shREST cells was harvested and subjected to gene expression microarray analysis. Of those genes that were up-regulated at least twofold upon REST knockdown, 93 were up-regulated in at least 2 cell lines, and 24 in all three cell lines (Figure 1B, Table S1). All of the 24 commonly upregulated genes are either known REST targets or putative REST targets as defined by the presence of a 17–33 bp REST binding element (RE1) within 12 kb of the coding region (Table S1). These genes formed the first iteration of our 24-gene signature (Figure 1B).

### REST function is lost in breast cancer

**Figure 2:**
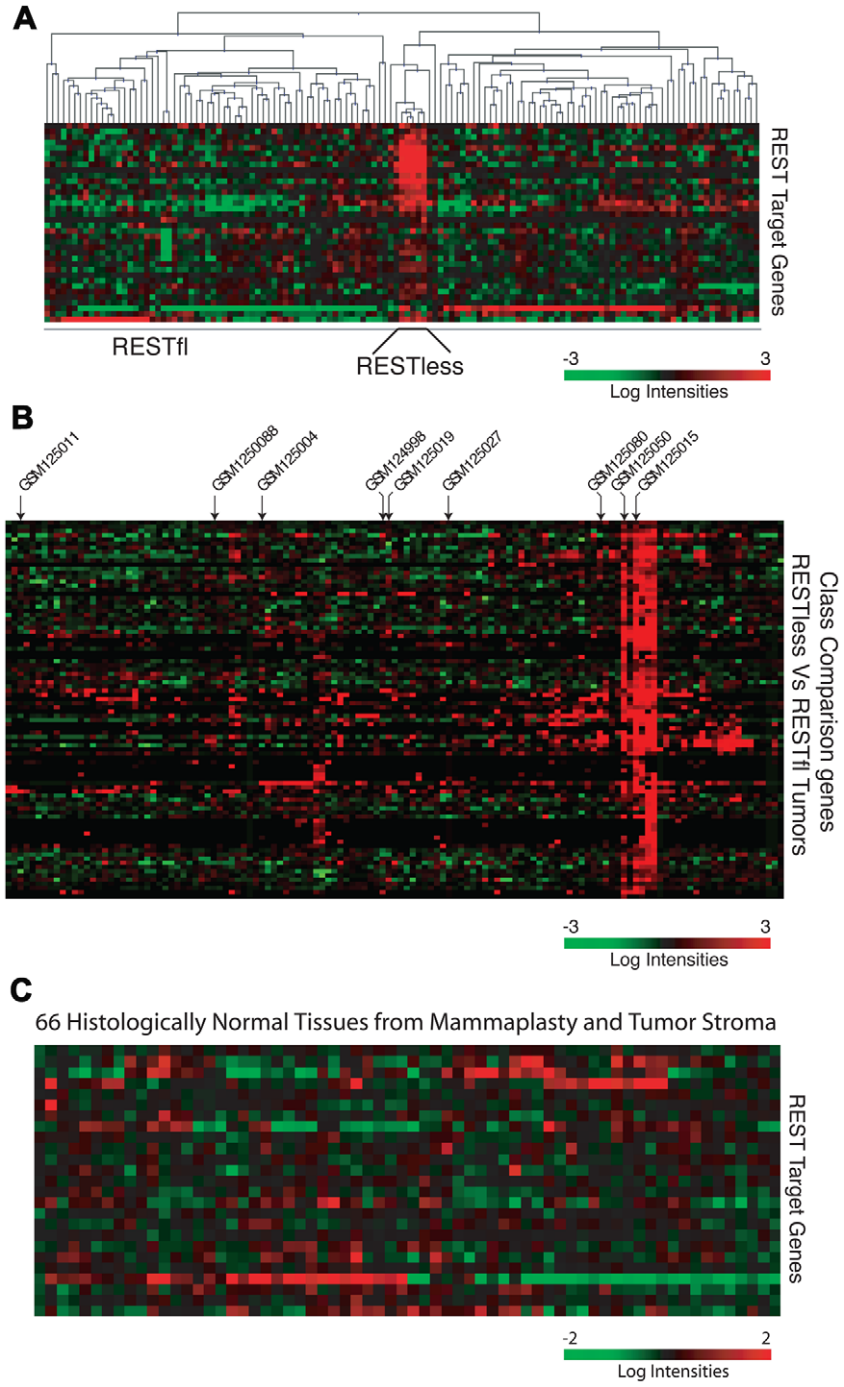
The 24-gene signature detects loss of REST function in breast tumors. (A) Hierarchical clustering analysis is performed on gene expression microarray data from 129 breast cancer tumors (GSE5460) across the 24-gene REST gene signature. Five tumors show a concerted overexpression of REST target genes, suggesting a loss of REST repression. (B) The expression of genes significantly upregulated in REST–less tumors (p<10^−7^) are shown, >85% are either known or putative REST target genes. Arrows indicate tumors from which RNA was available for further analysis. (C) The REST gene signature is applied 66 samples of normal mammary tissue obtained from mammaplasty and non-tumor stromal samples.

Using our 24-gene signature, we screened a microarray dataset containing 129 breast tumors [21] for loss of REST function. In a subset of tumor samples, we observed a concerted over-expression of REST target genes, and designated this group as REST–less (Figure 2A). To find other genes that showed this coordinated upregulation in REST–less tumors, we screened the microarray data for transcripts differentially expressed between REST–less and full-length REST (RESTfl for REST-full-length) expressing tumors using a random-variance t-test (Figure 2B). Of the 72 genes whose expression is most closely associated with the putative REST–less tumors (p<10^−7^), 63 (87.5%) either contain perfect consensus RE1 sites, are upregulated two-fold or greater upon REST knockdown, or were bound by REST in a genome-wide ChIP-Seq screen performed by Johnson *et al.* [4] (2), suggesting that they are direct targets of REST repression. To determine whether loss of REST function occurs exclusively in neoplastic mammary tissue, we used the 24-gene signature to screen 66 non-neoplastic mammary samples, half of which came from non-tumor bearing normal breast and half of which were adjacent normal stroma from a tumor-bearing breast [22]. We found no evidence of the 24-gene signature in any of the 66 stromal samples (Figure 2C), suggesting that the carcinoma cells carry this defect in tumors.

**Figure 3:**
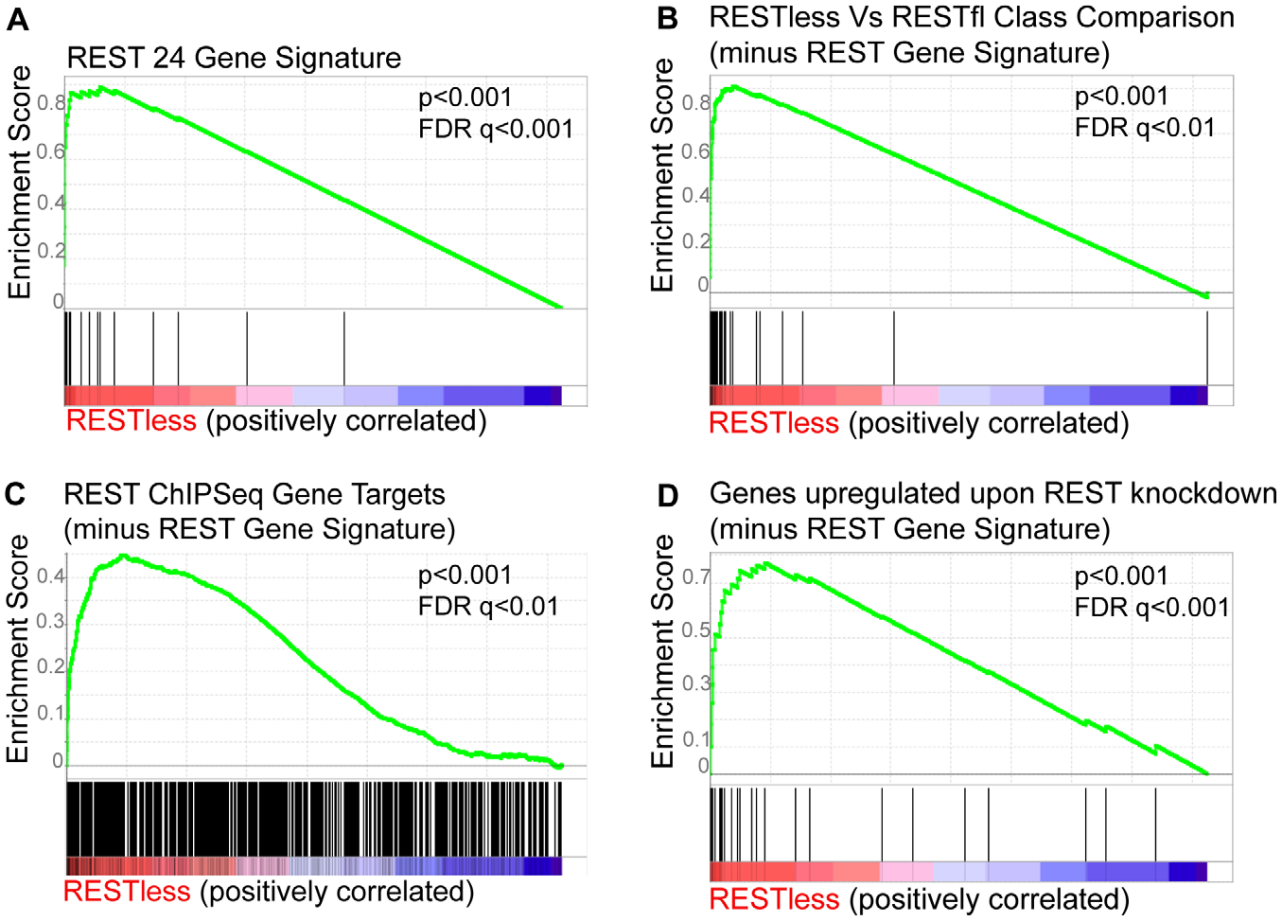
Gene set enrichment analysis of REST–less tumors. Gene Set Enrichment Analysis of the breast tumor dataset GSE5460 shows induction of REST target genes in REST–less tumors using three separate lists of experimentally defined REST target genes. (A) The “REST gene signature” 24-gene set comprised of 24 genes consistently upregulated at least two-fold upon REST knockdown in MCF10a, HEK-293 and T47D cell lines is enriched in REST–less tumors. (B) The list of genes identified in class comparison analysis of REST–less and RESTfl breast tumors are significantly enriched in REST–less tumors. (C) The “genes upregulated upon REST knockdown” gene set encompasses a wider range of REST targets, including all genes that were upregulated at least two-fold upon REST knockdown across the average of all three cell lines (minus the 24-gene signature). This geneset is also enriched in REST–less tumors. The ‘REST ChIPSeq’ gene list that is populated by genes identified as being bound by REST in Jurkat T-cells using ChIPSeq [4] (minus the 24-gene signature) is also significantly enriched in REST–less tumors.

To further test whether REST function was lost in REST–less tumors, we performed Gene Set Enrichment Analysis (GSEA) on the above breast tumors using the 24-gene signature (Figure 3A). This method compares the expression of a set of experimentally-defined REST target genes (termed “S”) between REST–less and RESTfl tumors, and assesses the relative enrichment of ‘S’ in either tumor group. The positive enrichment score obtained from these analyses, along with the low nominal P-value (p<0.001) and false discovery rate q-value (FDR q-value<0.001), are indicative of high level enrichment of REST target gene expression in the REST–less tumor subset not likely to occur by random chance. We also tested the gene set identified by the class comparison analysis (minus the 24-gene signature) using GSEA, and found that gene set to be strongly enriched in the REST–less tumor subgroup (Figure 3B). GSEA was also performed using an expanded list of genes that are at least two-fold over-expressed across the average of all three REST-knock-down cell lines (minus the 24-gene signature). This analysis found that REST–less tumors have a widespread and statistically significant loss of REST function (Figure 3C, nominal p-value<0.001, FDR q-value<0.001). Finally, we performed GSEA using an unbiased list of REST targets derived from a published ChIPSeq assay performed in a wholly different cell system, Jurkat T cells (Figure 3D)[4]. This unprejudiced approach also found that REST–less tumors, with respect to their RESTfl counterparts, are strongly enriched in the expression of REST target genes (nominal p-value<0.001, FDR q-value <0.01).

### REST mRNA levels are not altered in REST–less breast cancer

**Figure 4:**
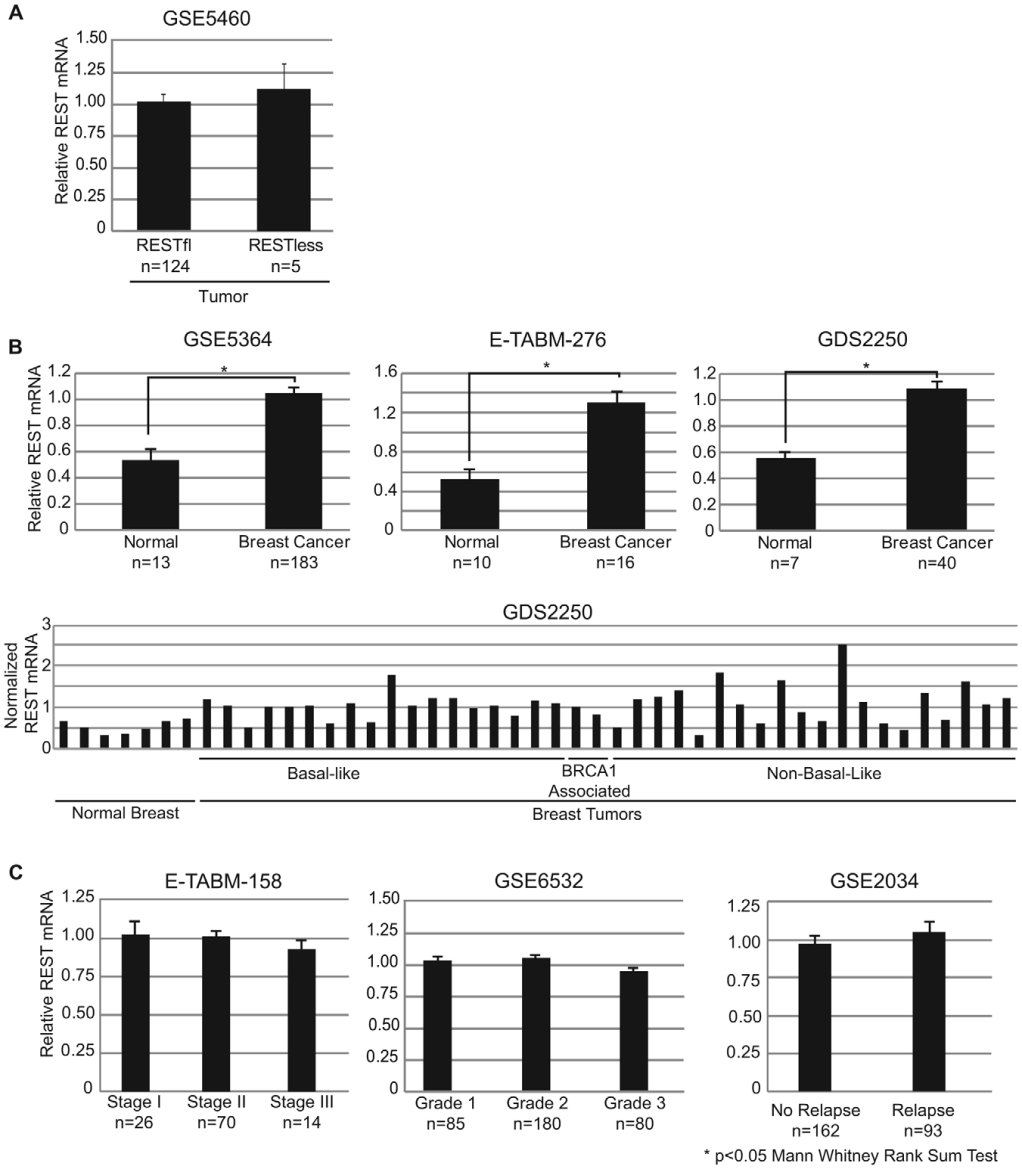
REST mRNA levels in breast tissue. (A) Mean REST mRNA levels were assessed in REST–less and RESTfl breast tumors from microarray dataset GSE5460. All error bars represent standard error. (B) Mean REST mRNA levels were compared in normal and tumor tissues across three independent datasets, all of which show a statistically significant increase in REST mRNA in tumor tissue, compared to normal (p<0.05 Mann Whitney rank sum test). Aggregate, and individual tumor data are displayed for dataset GDS2250, representing three distinct tumor types in addition to normal tissue. (C) REST mRNA data is presented from three independent datasets broken down by stratified by stage (E-TABM-158) grade (GSE6532) and eventual relapse (GSE2034). There is no significant difference in REST mRNA levels across any of these conditions.

We tested the possibility that the abrogation of REST function may be due to a loss of REST mRNA by comparing transcript levels in REST–less and RESTfl tumor samples. We saw no difference in REST transcript levels between the two tumor types (Figure 4A). Intriguingly, we noticed that REST mRNA levels were significantly higher in tumors than in normal tissue in three independent datasets representing over 250 normal and neoplastic tissue samples (Figure 4B). Furthermore, analysis of REST transcript levels in an additional 700 samples across multiple datasets found that REST mRNA levels are unaltered across tumors of increasing stage, grade, or likelihood to eventually relapse (Figure 4C).

### REST–less tumors show increased levels of the REST splice variant REST4

**Figure 5:**
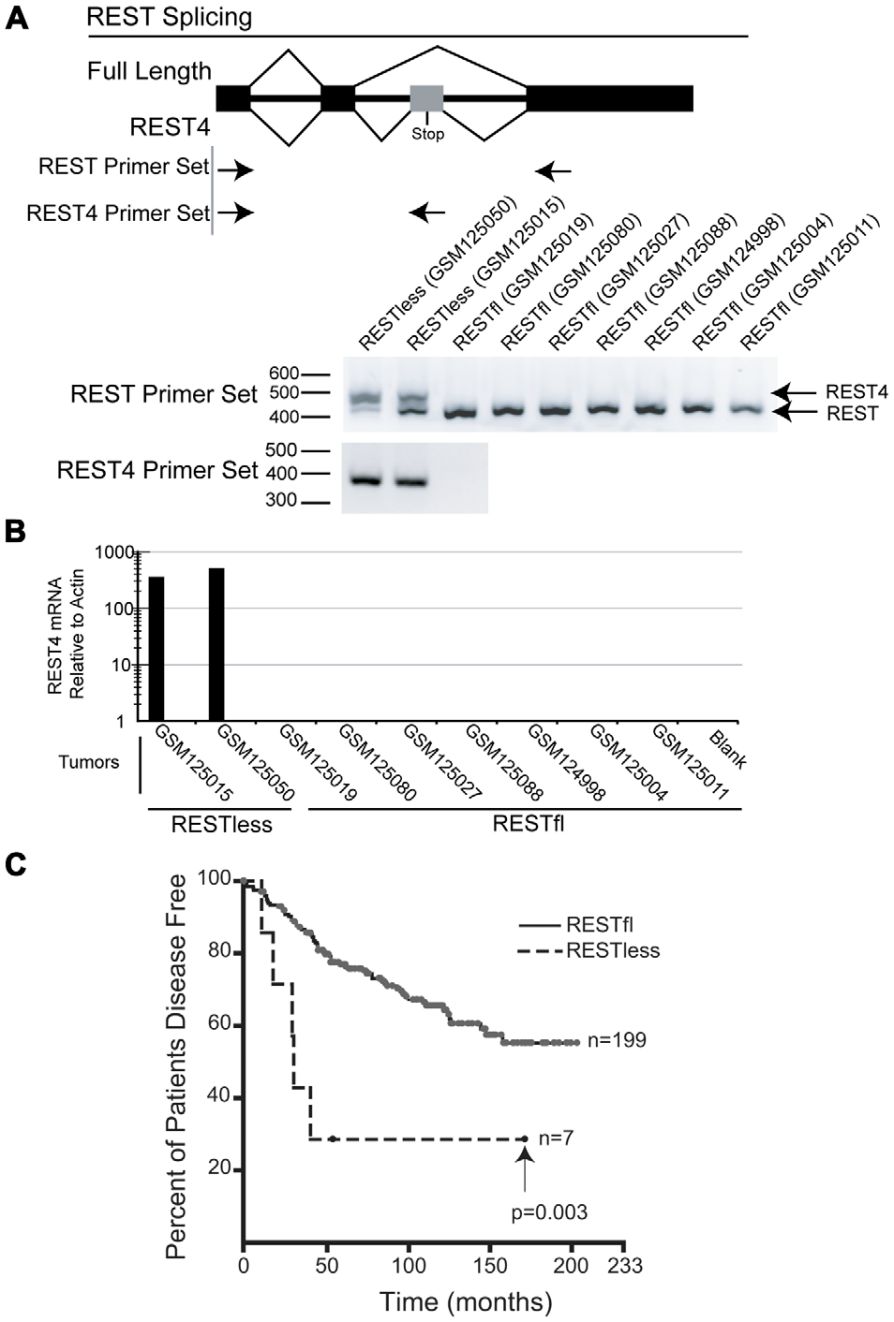
REST–less breast cancer tumors display high levels of REST4 splicing and poor prognosis. (A) Top panel: PCR primer screens for REST splicing in selected RNA from tumor samples indicated in Figure 2B: GSM124998, GSM125004, GSM125011, GSM125015, GSM125019, GSM125027, GSM125050, GSM125080 and GSM125088. Bottom panel: RNA was reverse-transcribed and PCR amplified with primers flanking the REST alternative intron/exon junction (REST primer set), showing alternative REST splicing only in the REST–less tumors. Additionally, selective PCR amplification of REST4 from tumor samples (using primers that target the REST4 50 bp exon (REST4 primer set)) demonstrated the presence of REST4 in the REST–less tumors, but not in any of the REST competent tumors. (B) Quantitative real-time RTPCR analysis of REST4 levels (relative to actin), in nine tumors represented in the microarray dataset GSE5460. REST4 mRNA, was detected in REST–less, but not RESTfl tumors after 35 cycles of amplification. (C) Patients with REST–less breast tumors in the superseries GSE6532, as defined by the 24-gene signature, show a significant decrease in their disease free survival with respect to their RESTfl counterparts (p<0.01).

Alternative splicing of REST to generate the truncated REST4 variant results in a protein with diminished DNA binding ability and loss of repressive function, and has been identified in the nervous system and in SCLC [8], [12], [23]–[25]. We therefore asked whether aberrant REST splicing could explain the loss of REST function in breast cancer. We were able to obtain RNA from two REST–less and seven RESTfl tumor samples which we amplified and interrogated for splicing by Reverse Transcription PCR using primers flanking the alternative REST4 exon. This analysis detected high levels of alternative splicing, resulting in the inclusion of the REST4 exon in REST–less tumors, which could not be detected in RESTfl tumors (Figure 5A). Selective amplification of REST4 using a primer placed in the REST4 exon confirmed the presence of the splice variant expression exclusively in the REST–less tumors (Figure 5A and 5B). Statistical analysis of these data using Fisher's exact test suggest that the presence of REST4 splicing in REST–less tumors alone was unlikely to have occurred by random chance (p<0.05). These data demonstrate that REST4 splicing is at least one mechanism by which full length REST may be lost in breast cancer.

We then used the 24-gene signature to classify the breast tumor superseries GSE6532 into REST–less and RESTfl tumors and determined how REST status associated with patient outcome (Figure 5C) [26]. This analysis shows that REST–less tumors identified by the REST gene signature manifest an extremely aggressive disease progression profile (logrank p-value<0.005). In this dataset, 57% of REST–less tumors recur within 3 years, compared to 13% of RESTfl tumors. Intriguingly, the REST–less tumors identified by gene signature show no additional relapse after 3.5 years, despite high levels of early aggression.

### Full-length REST is lost in breast cancer

**Figure 6:**
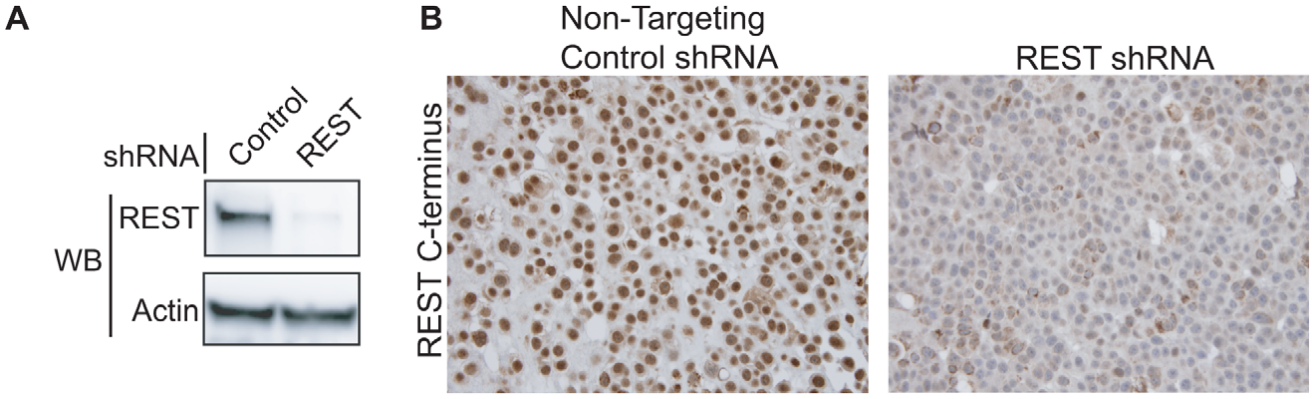
Testing the specificity of the REST C-terminal antibody. (A) MCF7 breast cancer cells were infected with lentivirus bearing either non-targeting control shRNA or REST targeting shRNA. REST knockdown was verified by western blot (Millipore antibody). Beta actin expression in shown as a loading control. (B) Paraffin-embedded MCF7 sections were stained with antibody to the REST C-terminus (Sigma) (stained brown), with hemetoxylin nuclear stain (stained blue). Control shRNA expressing cells show strong nuclear REST staining, while REST shRNA expressing cells show little to no nuclear REST staining.

To determine the frequency of REST protein loss in breast cancer, we developed an immunohistochemical (IHC) screen using an antibody directed to the C-terminus of REST (Atlas Antibodies, Stockholm). To test the specificity of the REST antibody, we utilized the REST-expressing breast cancer cell line MCF7. Cells were stably infected with lentiviruses bearing either non-targeting or REST-targeting shRNA (Figure 6A), then paraffin-embedded, sectioned and stained for REST C-terminus (Figure 6B). Non-targeting control shRNA expressing cells showed strong nuclear REST staining, whereas the REST knockdown cells show a severely depleted nuclear REST stain, suggesting specificity for the antibody at levels of REST observed in breast cancer cells. REST4 and the truncated form of REST identified as a SNP in colon cancer [14] are not recognized by this antibody, allowing us to identify tumors that lack full-length REST.

**Figure 7:**
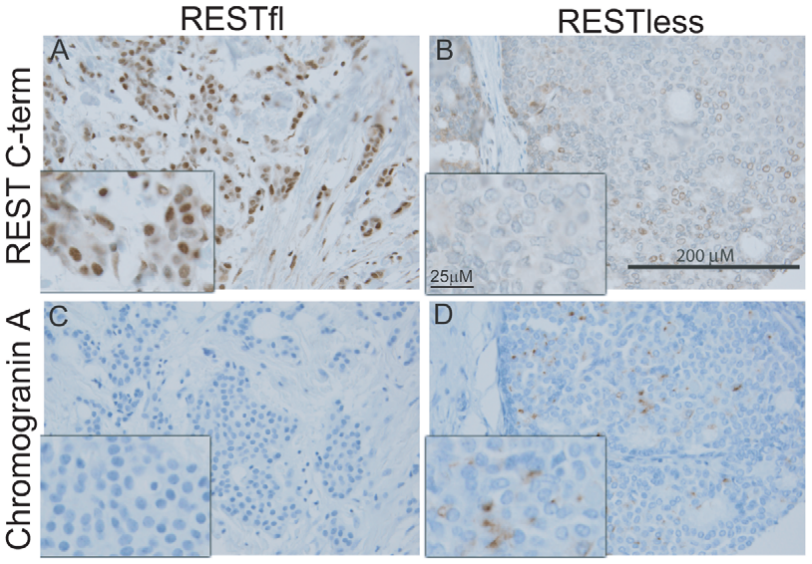
Immunohistochemical screen for functional REST in breast tumors. (A,B) Paraffin embedded breast tumor sections were immunohistochemically labeled with an antibody to the C-terminus of REST. Inset image is enlarged 2.5× to show detail. (A) Strong nuclear REST labeling in the majority of tumor cells (brown signal). (B) Weak or absent labeling for REST in REST–less tumor. (C,D) Paraffin embedded breast tumor sections were stained for the protein product of the REST target gene chromogranin-A (*CHGA*). (C) No CGA labeling detected in RESTfl tumor (blue hematoxylin stain highlights nuclei of carcinoma and stroma cells). (D) Moderate to strong labeling for CGA in a subset of carcinoma cells. The brown label (CGA) does not co-localize with the nuclear hematoxylin counterstain, suggesting a non-nuclear location. REST–less tumors were significantly enriched in chromogranin-A staining (D, p<0.001).

**Figure 8:**
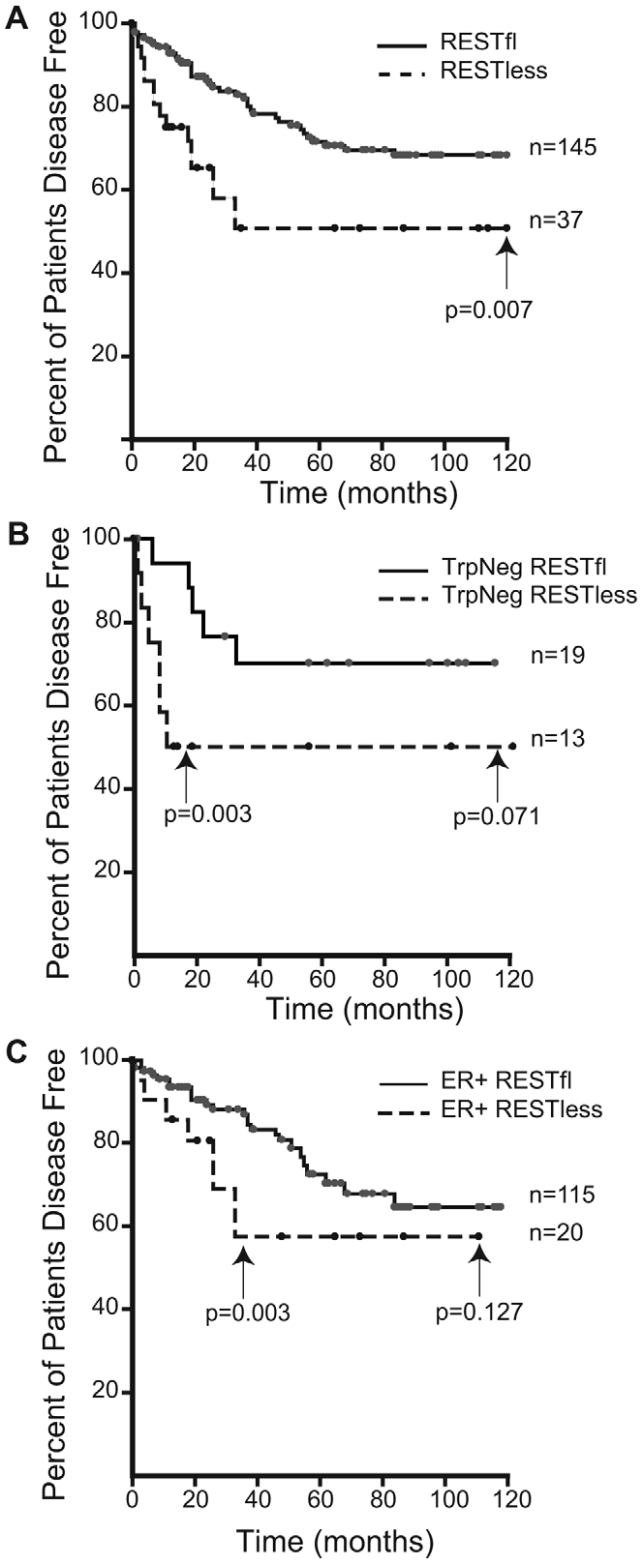
Outcome data for patients with REST–less and RESTfl breast tumors. (A) Patients with REST–less breast tumors show significantly decreased disease free survival time (p = 0.007, n = 182), and increased incidence of relapse (p = 0.054, n = 182), particularly in the first three years post diagnosis. (B) Patients with REST–less estrogen receptor/progesterone receptor/HER2 negative (TrpNeg) breast cancer show a similar pattern of high tumor aggression in the first year post-diagnosis, relative to their RESTfl counterparts. (C) Patients with REST–less estrogen receptor (ER) positive tumors show high levels of disease aggression in the first 3 years post diagnosis.

Immunohistochemical analysis of 182 breast tumors in a tissue microarray confirmed the lack of full-length (functional) REST predicted by the REST4 splicing in 37 (20.3%) tumor samples (Figure 7B). Additionally, these REST–less tumors showed a significant enrichment in staining for the ectopic expression of the REST target gene chromogranin-A (*CHGA* Enterez GeneID 1113), consistent with a loss of REST repression (Figure 7D, p<0.001). Importantly, we found that REST–less status correlated significantly with poor disease-free survival (Figure 8A, p = 0.007), and the average time to relapse for REST–less tumors (14 months) is less than half of that for RESTfl tumors (35.9 months p = 0.0217). REST–less tumors from this cohort have significantly increased tumor size and lymph node involvement, alongside several other markers of aggressive, treatment-resistant breast cancers summarized in Table 1 . Patients with REST–less triple negative (TN) tumors (Estrogen Receptor (ER)/Progesterone Receptor/HER2-) endure significantly greater disease recurrence within 2 years than TN/RESTfl patients (50% versus 20% recurrence, p = 0.044, n = 32) (Figure 8B). Patients with REST–less ER+ breast tumors are also more prone to relapse in the first 3 years (Figure 8C, p = 0.003, n = 135). Strikingly, 100% of disease recurrence events for patients with REST–less tumors occurred in the first 36 months, compared to just 61% of all recurrence events for patients with RESTfl tumors. After 3 years there were no additional recurrences of REST–less tumors. This suggests that the loss of REST leads to a more uniquely aggressive disease that is most likely to recur within 3 years of diagnosis with no observed occurrence thereafter. Importantly, the IHC staining for REST C-terminus has the ability to identify tumors that have lost full-length REST via multiple mechanisms, including REST4 splicing, ubiquitin-mediated degradation or truncating mutation, as has been seen in colon cancer [14].

**Table 1:** Patient outcome and tumor data corresponding to 182 paraffin embedded breast tumors stained with antibody to the REST C-terminus.

		% Chromogranin Positive	% HER2 Positive	Grade	Size	Time To Relapse	Patient Age	Nodal Number	Percent Relapse
All Tumors	REST–less (n = 37)	**10.8% (0.0007)**	13.5% (0.496)	**2.41+/−0.13 (0.0269)**	**3.88+/−0.39 (0.0012)**	**14.0+/−1.8 (0.0217)**	**53.4+/−2.19 (0.0494)**	**4.8+/−1.2 (0.020)**	43% (0.054)
	RESTfl (n = 145)	**0.7%**	9.7%	**2.07+/−0.07**	**2.65+/−0.16**	**35.9+/−3.02**	**58.3+/−1.11**	**2.6+/−0.4**	27.0%
ER+Tumors	REST–less (n = 20)	**15% (0.0007)**	**10% (0.0164)**	1.95+/−0.18 (0.45)	**3.49+/−0.55 (0.0142)**	17.3+/−2.6 (0.127)	55.45+/−3.3 (0.0716)	3.95+/−1 (0.127)	35% (0.161)
	RESTfl (n = 115)	0.9%	**2.6%**	1.86+/−0.07	**2.59+/−0.17**	42.4+/−3.82	60.00+/−1.2	2.3+/−0.4	23.0%
Triple Neg Tumors	REST–less (n = 13)	7.7% (0.233)		2.92+/−0.1 (0.217)	3.45+/−0.3 (0.0568)	**5+/−0.88 (0.0109)**	50.8+/−3.5 (0.0895)	6.92+/−2.4 (0.168)	46% (0.26)
	RESTfl (n = 19)	0.0%		3.00+/−0	2.47+/−0.4	**16.6+/−1.9**	53.0+/−2.8	3.47+/−1.1	26.0%

Mean Values +/− Standard Error (Pearson p-value) Bold values indicate statistical significance (p<0.05).

Triple Neg tumors are estrogen receptor, progesterone receptor, and HER2 negative.

## Discussion

In this study we demonstrate that REST function is lost in a fraction of breast tumors. Loss of REST is defined by either the presence of a 24 gene signature in tumors or by loss of immunohistochemical stain with a antibody raised to the C-terminus of REST. REST–less tumors (as determined by either IHC or 24-gene signature) are associated with an aggressive, rapid recurrence prognosis. At this time it is not known whether REST–less tumors defined by gene signature represent a subset of those determined to be REST–less by IHC or are a distinct group of malignancies. Answering this question will allow for the elucidation of the suite of mechanisms by which REST function can be lost in breast cancer and will require the analysis of patient cohorts that have associated patient outcome data, tissue micro-arrays as well as gene expression data.

### A 24-gene signature identifies a distinct cohort of breast tumors expressing REST4

We have developed two complementary methods of screening tissue and tumors for their levels of functional REST. For samples associated with microarray data, a signature of 24 genes indicates loss of functional REST. Histological sections can be stained for the C-terminus of REST in combination with the REST target gene, chromogranin-A.

It is known that post-transcriptional regulation of REST occurs during neuronal differentiation and during oncogenic transformation such that protein levels are significantly reduced in the absence of altered mRNA levels [15], [16], [27]. These observations support our findings that REST function cannot be directly measured by its mRNA levels in breast tumor oligonucleotide arrays. However, the development of a gene signature for loss of REST *in vitro* allowed for the identification of a class of REST–less breast tumors. Analysis of tumors so characterized showed that REST was aberrantly spliced to generate the REST4 truncated variant. REST4 was originally identified in the hippocampii of mice after kainic acid induced seizures [23] and results in the inclusion of an alternative exon (exon ‘N’ which is 62 bp in humans) between exons V and VI of the REST transcript that encoded a stop codon and places the downstream mRNA out of frame. REST4 transcript is therefore 62 bp longer than REST transcript but REST4 protein possesses only 5 of the 8 zinc fingers in the DNA binding domain and lacks the C-terminal repression domain [6], [23]. The conversion of REST to REST4 in the hippocampus was associated with de-repression of the REST target gene BDNF.

Importantly, REST4 splicing is regulated by cell signaling pathways and in the case of seizure models, the alternative splicing of REST occurs within a limited time window before full-length, functional REST transcript splicing is restored. Indeed, in SCLC cell lines reintroduction of full-length REST induces apoptosis. Similarly, reintroduction of full-length REST into REST–less colon cancer cell lines restores anoikis, blocking anchorage-independent cell growth [14]. Therefore, we believe that the signaling pathways regulating potentially reversible REST4 splicing represent an attractive pharmacological opportunity for combating REST–less breast cancer.

### Lack of REST function correlates with poor outcome

To better understand the disease course taken by REST–less tumors we took advantage of the fact that REST4 lacks the C-terminus. This allowed us to verify loss of full-length REST via an immunohistochemical screen utilizing an antibody raised to the C-terminus of REST, which will not detect REST4, degraded REST, or any previously characterized truncated mutant of REST. IHC on a panel of 182 tumor samples with associated outcome data showed that patients with REST–less tumors experience a 20% reduction in disease free survival when compared to their RESTfl counterparts over 10 years (Figure 8A). The majority of the outcome disparity between patients with REST–less and RESTfl tumors occurs in the first three years post-diagnosis in REST–less tumors identified by either the gene signature or IHC method. In both cases, at least 50% of patients with REST–less tumors showed recurrence within three years. By comparison, less than 20% of patients with RESTfl tumors showed recurrence within three years in both datasets examined. Tumors that stain negative for the REST C-terminus correlate strongly with decreased time to disease recurrence, increased tumor size, and a higher number of lymph node metastases, all of which indicate a more aggressive disease course (Table 1).

Remarkably, a subset of tumors from all histological classes of breast cancer stained negative for REST C-terminus, and all classes showed a worse prognosis without REST. REST–less triple negative tumors showed a particularly aggressive disease course. Of the 32 triple negative tumors screened, 13 were found to be REST–less, six (46%) of which recurred in the first 12 months post-diagnosis, compared to just 1 of the 19 (5%) RESTfl triple negative tumors (p = 0.003). However, no triple negative REST–less tumor recurred after 12 months in 10 years of patient outcome data. ER+ REST–less tumors showed a similar pattern of early recurrence, wherein 8 of 21 (38%) patients saw disease recurrence in the first 36 months, compared to just 11% of ER+ RESTfl patients (p = 0.003). Thereafter, none of the remaining 13 disease-free patients with ER+ REST–less tumors experienced recurrence, compared to 12 of the 102 remaining disease-free ER+ RESTfl patients. These data suggest that REST–less tumors represent a distinct, aggressive subset of breast tumors with a unique disease course.

In their recent paper, Reddy *et al.* examine REST levels in 19 samples of normal and neoplastic breast tissues. Using 3 control tissue samples and 16 tumors spread among 3 tumor stages, they suggest that REST protein and mRNA levels undergo a stepwise, statistically significant decrease with each increasing tumor stage [28]. We examined gene expression in over 250 samples of tumor and normal tissue in three different publicly available databases and find that breast tumors express REST mRNA at levels at or above those in normal tissue. We did not find a single tumor with REST mRNA levels lower than that of the control tissue. After further analysis of over 700 additional tumors of varying grade, stage, and eventual recurrence, we find that REST mRNA levels are invariant with any of these conventional measures of tumor aggression (Figure 4C). Reddy *et al.* reported that they did not observe any alternate splice variants in the tumor samples in which they find REST to be lost (Reddy *et al*.– data not shown). However, we have found alternative REST splicing in tumors where REST function is lost (Figure 5). The reason for these discrepancies is not clear at this time, but understanding the breadth and mechanism of REST loss in breast cancer is key to understanding its role in the disease, and thus should be based on robust and consistent data.

Breast cancer is a heterogeneous disease, with histologically identical tumors often displaying very different disease courses and responses to treatment. Unsupervised hierarchical clustering of breast tumors based on their transcriptional profiles has uncovered at least five unique subtypes of breast cancer [29]–[31]. Additionally, at least two different gene signature based molecular diagnostics (Oncotype DX and Mammaprint) are currently in use clinically to help physicians and patients predict risk of metastasis and response to treatment [32]. Importantly, the 24-gene REST gene signature does not have any genes in common with any of the above genesets, and is unique in that the cause of the gene signature is known. Understanding the origin of this unique breast cancer subtype is important, as it may lead to the development of REST-targeted therapies.

In summary, we show here that REST–less tumors represent a distinct, aggressive subset of breast tumors with a unique disease course. We find that the REST–less gene signature is closely associated with the presence of the truncated REST4 splice variant and that REST status is an important predictor of poor prognosis that correlates with increased lymph node metastasis and early disease recurrence.

## Materials and Methods

### Cell culture conditions

All cells were grown in 5% CO_2_ at 37°C. HEK-293 and MCF7 cells were grown in DMEM with 4.5 g/L glucose, 2 mM L-Glutamine, and 10% fetal bovine serum from HyClone (Logan, UT). T47D cells were grown in RPMI with L-glutamine, 10 ug/mL insulin, and 10% fetal bovine serum. MCF10a cells were grown in DMEM/F12 with 5% horse serum, 10 mg/mL insulin, 20 ng/mL epidermal growth factor, and 0.5 mg/mL hydrocortisone.

### Lentiviral knockdown

Stable REST knockdown in HEK-293, T47D and MCF10a cells for microarray analysis was achieved using a Dharmacon SMARTvector lentiviral shRNA delivery system as per manufacturer's instructions. Briefly, cells were infected in the presence of 8 mg/mL polybrene at an MOI of 5 with virus expressing a non-targeting control or REST shRNA. Puromycin selection was begun 48 hours after infection and maintained during cell expansion and experimentation. SMARTvector Lentiviral Particles (catalog #SH-042194-01-25) towards REST targeted the sequence GCAAACACCTCAATCGCCA, Non-Targeting SMARTvector shRNA Lentiviral particles (catalog #S-005000-01) were used as an infection control.

### Microarray data generation and processing

RNA was extracted using TRIzol (Invitrogen) as per the manufacturer's instructions from four independent plates of each cell line T47D, HEK-293 and MCF10a, with two biological replicates of cells expressing REST shRNA and another two biological replicates expressing a non-targeting control shRNA.

All RNA reverse transcription, amplification and hybridizations were performed by the University of Wisconsin-Madison Gene Expression Center (http://biotech.wisc.edu/GEC/). RNA integrity and quality were assessed by comparing 28S/18S rRNA ratio using Agilent RNANano6000 chips on an Agilent 2100 Bioanalyzer. First and second strand cDNA synthesis steps, followed by *in vitro* transcription, were performed using the Ambion Amino Allyl Messageamp II kit. Cy3 and Cy5 (Amersham) dyes were coupled to the aRNA, with each fluorophore labeling a separate biological replicate, before fragmentation and dual hybridization to Nimblegen HG18 60mer 385k Gene Expression Arrays (Nimblegen, Cat #A4542-00-01). For dual hybridization, shControl and shREST samples from the same cell line were competitively hybridized. Arrays were scanned on an Axon4000B and the gene expression data was extracted, and RMA normalized using software provided by Nimblegen.

Bioinformatic analyses on the microarray data were performed using BRB-ArrayTools v3.7 (developed by Dr. Richard Simon and BRB-ArrayTools Development Team) and MultiExperiment Viewer 4.5.1. Tumor gene expression data were obtained from the NCBI Gene Expression Omnibus, and are identified by their GEO dataset record number. Dataset E-TABM-276 was downloaded from the Ensembl ArrayExpress. Analysis of dataset GSE6532 was performed to determine the aggressiveness of tumors identified as being REST–less using the gene signature method. All samples from this dataset that included information on duration of relapse free survival as well as relapse event information were included in our analysis, for a total of 206 outcome-associated tumors. Hierarchical clustering was performed using a one-minus correlation metric with average linkage over centered genes. Cluster diagrams were produced using BRB Arraytools, Cluster 3.0 and TreeView software. Class comparison analysis between REST–less and RESTfl tumors was performed using a random variance model for univariate tests with a significance threshold of p<10^−7^ to reduce false positives.

Gene Set Enrichment Analysis (GSEA) was performed using the GSEA program provided by the Broad Institute. GSEA compares the expression of a set of experimentally defined REST target genes (termed “S”) between REST–less and RESTfl tumors, and assesses the relative enrichment of S in either tumor group. The positive enrichment scores obtained from these analyses are indicative of high level enrichment of REST target gene expression in the REST–less tumor subset. To determine whether this enrichment could arise by random chance, 1,000 permutations of the above analysis were performed with each permutation randomly assigning tumors as being either REST–less or RESTfl and then assessing enrichment in each group for S. The fraction of permutations that result in an enrichment for REST target genes in the randomly assigned “REST–less” tumor group serves as an estimate of statistical significance and is presented as a nominal p-value (Subramanian et al.PNAS 2005). Similarly, the false discovery rate (FDR) q-value is a measure of the likelihood that a geneset S may be a false positive, but this statistic is controlled for gene set size and may therefore be compared between multiple genesets with normalized enrichment scores. The list of REST target genes identified in the Johnson *et al.* ChIP-Seq were chosen on the basis of their enrichment in both experiments 1 and 2 in a region carrying an RE1 site with a p-value of <10^−4^. Genesets used in GSEA analysis are available as Table S3.

### Amplifying REST4 from tumor RNA

The work with human tumor RNA was found to be exempt from institutional review by the UW institutional review board (IRB). Tumor RNA was amplified in duplicate according to the Affymetrix manual on Eukaryotic Target Preparation, Section 2.1 (https://www.affymetrix.com/support/downloads/manuals/expression_s21_manual.pdf). Briefly, a first strand of cDNA was generated from 100 ng of tumor RNA using T7-oligo(dT) primers, incubated at 70°C for 10 minutes, followed by 2 minutes on ice. A first-strand reaction mix with dNTPs, DTT and SuperScript III Reverse Transcriptase (Invitrogen) was added to each sample and incubated for an hour at 42°C, then transferred to ice. The second strand of cDNA synthesis was performed using a second-strand reaction mix, dNTPs, *E. coli* DNA ligase, *E. coli* DNA polymerase, and RNase H, and was incubated at 16°C for 2 hours. T4 DNA polymerase was added for the final 5 minutes of this incubation period, generating full-length double-stranded cDNA. The cDNA was purified on MinElute PCR purification columns (Qiagen) as per manufacturer's instructions. The Megascript T7 RNA polymerase kit (Ambion) was used to generate aRNA from the tumor cDNA as per the manufacturer's instructions. The aRNA was purified from the reaction mix using Trizol (Invitrogen) as per the manufacturer's instructions, and 500 ng of tumor aRNA was used to generate tumor cDNA using SuperScript III RT Kit (Invitrogen) as per manufacturer's instructions. This cDNA then served as a template for the PCR amplification of REST using Pfx Platinum high fidelity polymerase (Invitrogen) as per manufacturer's instructions.

Quantitative real-time PCR analysis of REST 4 was performed using a 5 ng aRNA equivalent of cDNA, the SYBR qRT-PCR System (Takara) and hREST4 Forward and hREST SV Region Reverse primers over 35 cycles.

Primers used to amplify the exon junctions surrounding introns 1 and 2:

hREST SV region forward: GAGCGAGTATCACTGGAGGAAACATTT

hREST SV region reverse: ATAGTCACATACAGGGCAATTGAACTGC

Primers used to amplify REST4:

hREST4 forward (Used with hREST SV reverse): CATTCAGTGGGGTATGGATACC

hREST4 reverse (Used with hREST SV forward): GCTTCTCACCCATCTAGATCAC

### Immunohistochemistry on tissue microarray and outcome analysis

Four-micron sections of previously characterized tissue microarrays, which contain duplicate tissue cores from 207 human breast carcinomas, were used for the labeling experiments [33]. Chromogranin A labeling was performed on an automated Ventana instrument (Ventana Medical Systems, Tucson, AZ). After a standard deparaffinization procedure, epitope retrieval was performed with CC1 high-pH buffer (Ventana). In the automated protocol, the prediluted anti-chromogranin A antibody (Clone LK2H10, Ventana) was added for 32 minutes at 42°C. After addition of a universal secondary antibody, target detection was accomplished with an indirect biotin-avidin-peroxidase procedure. Labeling for REST was performed on a Lab Vision Autostainer 360. After deparaffinization, heat-induced epitope retrieval with citrate buffer and endogenous peroxidase inhibition, the slides were blocked with Background Sniper (Biocare Medical). The sections were then incubated with rabbit anti-REST antibody (HPA006079, Sigma-Aldrich) at a concentration of 0.5 µg/ml for 60 minutes. After washing, the Mach 3 detection system (Biocare Medical) was applied. The labeling reaction was manually scored by a board-certified pathologist (AF) for the cytoplasmic and nuclear carcinoma cell compartments, using the method described by Harvey and colleagues [34].

Kaplan Meier plots were generated using SigmaStat 3.0, and Kaplan Meier curves were compared using the logrank test. We report as significant only those curves that have reached a level of p≤0.05. Correlations between REST status and disease parameters were tested using Pearsons Chi-squared test, and are reported as significant p≤0.05.

## Supporting Information

Table S1 This table lists genes that show a >2 fold upregulation following REST knockdown, as determined by oligonucleotide microarray in 3 cell lines: HEK-293, MCF10a, and T47D cells.
(0.10 MB XLS)

Table S2 This table describes the gene list determined to be associated with RESTless tumors in dataset GSE5460 by class comparison. This list of genes represents all of the genes identified as more highly expressed in RESTless tumors with respect to RESTfl tumors in the GSE5460 dataset (p<e-7) by using a random variance model for univariate tests in BRB Arraytools v3.7. Of the 72 genes in this list, 63 are putative REST targets, as defined by a consensus RE1, positive ChIP Seq signal, or a two-fold change upon REST knockdown in at least one of three cell lines.
(0.08 MB XLS)

Table S3 This table provides the gene sets used in gene set enrichment analysis of dataset GSE5460.
(0.06 MB XLS)
